# Assessing Nesting Status of Green Turtles, *Chelonia Mydas* in Perak, Malaysia

**DOI:** 10.21315/tlsr2018.29.1.11

**Published:** 2018-03-02

**Authors:** Sarahaizad Mohd Salleh, Shahrul Anuar Mohd Sah, Ahmed Jalal Khan Chowdhury

**Affiliations:** 1 School of Biological Sciences, Universiti Sains Malaysia, 11800 USM Pulau Pinang, Malaysia; 2 Centre for Marine and Coastal Studies (CEMACS), Universiti Sains Malaysia, 11800 USM Pulau Pinang, Malaysia; 3 Department of Marine Science, Kulliyyah of Science, International Islamic University Malaysia, 25200 Kuantan, Pahang, Malaysia; 4 Institute of Oceanography and Maritime Studies (INOCEM), Kulliyyah of Science, International Islamic University Malaysia, 25200 Kuantan, Pahang, Malaysia

**Keywords:** Conservation, Green Turtle, Nest, Survival Hatchling

## Abstract

The nesting of green turtle (*Chelonia mydas*) was monitored from 1998 untill 2013 along the beaches of Pasir Panjang, Segari, Perak. The objective of the study is to assess the nesting status of green turtles in Perak, Peninsular Malaysia in terms of total nests, eggs, survival hatchings, and density of visitors. A total number of green turtle nests found for 16 years were 1,019 nests and varied from 10 to 220 nests per year. Meanwhile, the sum of eggs collected for 16 years were 107,820 eggs, and varied from 553 to 20,881 eggs per year. The temporal pattern of nesting indicates year-round nesting in Perak in most years within the 16 years period. The peak season of nesting was estimated to occur between May and June. Survival hatchlings varied from 23.33% (2,071 hatchlings) to 55.03% (5,018 hatchlings) from 1998 to 2013. The density of visitors was not uniformly distributed among the years, and shows a sign of decline especially from 2006 onwards. This publication provides basic knowledge of green turtle nesting population in Perak, and would be helpful in upgrading the conservation program in Malaysia. In future, we hope 1) for an increase in manpower to obtain accurate nesting records along the nesting beaches during nocturnal survey and, 2) to include the breeding biology data such as nest placement, emergence hour, and morphological characteristics of green turtle.

## Abstract (ru)

Sarang penyu agar (*Chelonia mydas*) di pantau dari tahun 1998 hingga 2013 di sepanjang pantai Pasir Panjang, Segari, Perak. Objektif kajian ini ada untuk melihat status bersarang penyu agar di Perak, Semenanjung Malaysia dari sudut kajian jumlah sarang, telur, survival anak penyu, dan kepadatan pelawat. Jumlah sarang penyu agar yang di jumpai dalam tempoh 16 tahun adalah sebanyak 1,019 sarang, dan berbeza dari 10 hingga 220 sarang setiap tahun. Sementara itu, jumlah telur yang dikutip dalam tempoh 16 tahun adalah sebanyak 107,820 telur, dan berbeza dari 553 hingga 20,881 telur setiap tahun. Corak temporal menunjukkan Perak mempunyai taburan sarang di sepanjang tahun di kebanyakan tahun dalam tempoh 16 tahun. Musim kemuncak waktu bersarang dijangkakan berlaku di antara Mei dan Jun. Survival anak penyu berbeza dari 23.33% (2,071 anak penyu) hingga 55.03% (5,018 anak penyu) dari tahun 1998 hingga 2013. Kepadatan pelawat adalah tidak seragam antara tahun, dan menunjukkan penurunan terutamanya dari tahun 2006 dan ke atas. Penerbitan ini mengetengahkan ilmu asas berkaitan populasi sarang penyu agar di Perak, dan mungkin berguna ke arah meningkatkan program pemuliharaan di Malaysia. Di masa hadapan, kami mengharapkan 1) peningkatan jumlah tenaga pekerja semasa tinjauan di waktu malam di sepanjang pantai bersarang untuk ketepatan rekod catatan, dan 2) mengetengahkan maklumat biologi pembiakan seperti lokasi penempatan sarang, masa pendaratan, dan ciri-ciri morfologi penyu agar.

## INTRODUCTION

In Malaysia, the green turtle is widely distributed, with the prominent nesting population being in Sabah (specifically at Turtle Islands Park), Sarawak (specifically at Talang Satang National Park), and Terengganu (specifically at Redang Island and Setiu). On the west coast of Peninsular Malaysia, there are three highlighted marine turtle nesting beaches facing the Straits of Malacca; these are Penang Island, Perak, and Melaka. The green turtle majorly resides in Penang Island (specifically in Kerachut and Teluk Kampi; Sarahaizad *et al.* 2012a) and Perak (Pantai Remis; Chan 2006) with minor landing of olive ridley. Meanwhile, the hawksbill species majorly resides in Melaka with no records of green turtle nesting (Mortimer *et al.* 1993). It was previously mentioned that only the green and olive ridley species can be found in Perak state (Pusat Pengurusan Penyu, Segari, Perak Darul Ridzuan 2007), which is similar to the adjacent state, Penang Island. However, the exact population in Perak is still vague due to minimal published information, thus difficult to compare both the states. In comparing to the other nesting sites in Malaysia, the population status of marine turtle was stated in terms of nesting per years, and published by Mortimer *et al*. 1993 (Melaka), Setiu, Terengganu (Aini Hasanah *et al.* 2014), Redang Island (Chan 2010), Penang Island (Sarahaizad *et al*. 2012a), Sabah (Chan *et al*. 1999) and Sarawak (Leh 1994). Due to the above reason, the rational of this study is to observe on the nesting status in Perak with full co-operation by the Department of Fisheries. In addition, we also would like to observe the current conservation plan implemented by the Department of Fisheries in Turtle Management Centre, Pasir Panjang (Perak) and to provide recommendations for further improvement. The objective of the study is to assess the nesting status of the green turtle in Perak, Peninsular Malaysia in terms of nests, eggs, survival hatchings and density of visitors. Currently, the green turtle is listed as Endangered or Vulnerable in the IUCN Red List of Threatened Species worldwide (Mortimer & Donnelly 2008).

## MATERIALS AND METHODS

### Study Sites

Perak is one of the Malaysian States located on the west coast of Peninsular Malaysia. Perak borders Kedah at the north, Kelantan and Pahang to the east, and Selangor to the south, and faces the Straits of Malacca to the south. Pasir Panjang beach in Segari is the main turtle landing area in Perak (Pusat Pengurusan Penyu, Segari, Perak Darul Ridzuan 2007). The research was performed at Pasir Panjang beach, Segari (located in Perak), with the help from the staff of Turtle Management Centre.

Realising the threat of extinction to the marine turtle species, the area of Pasir Panjang beach in Segari was develop as a research centre for marine turtles. The Turtle Management Centre (Latitude: 4.3470, Longitude: 100.5860) was initiated in 1990, developed under the management of Department of Fisheries. The centre is located on a 2.4-hectare area and has interpretative pond and exhibition gallery to disseminate information to create awareness amongst public. To date, this Turtle Management Centre has contributed in enhancing the turtle population through hatching and release programs, gathering information through research and monitoring, conducting public awareness events, and spreading the conservation to the young generations. The information of Turtle Management Centre was obtained from a booklet published by Pusat Pengurusan Penyu, Segari, Perak Darul Ridzuan (2007).

### Beach Patrol

**Figure 1:**
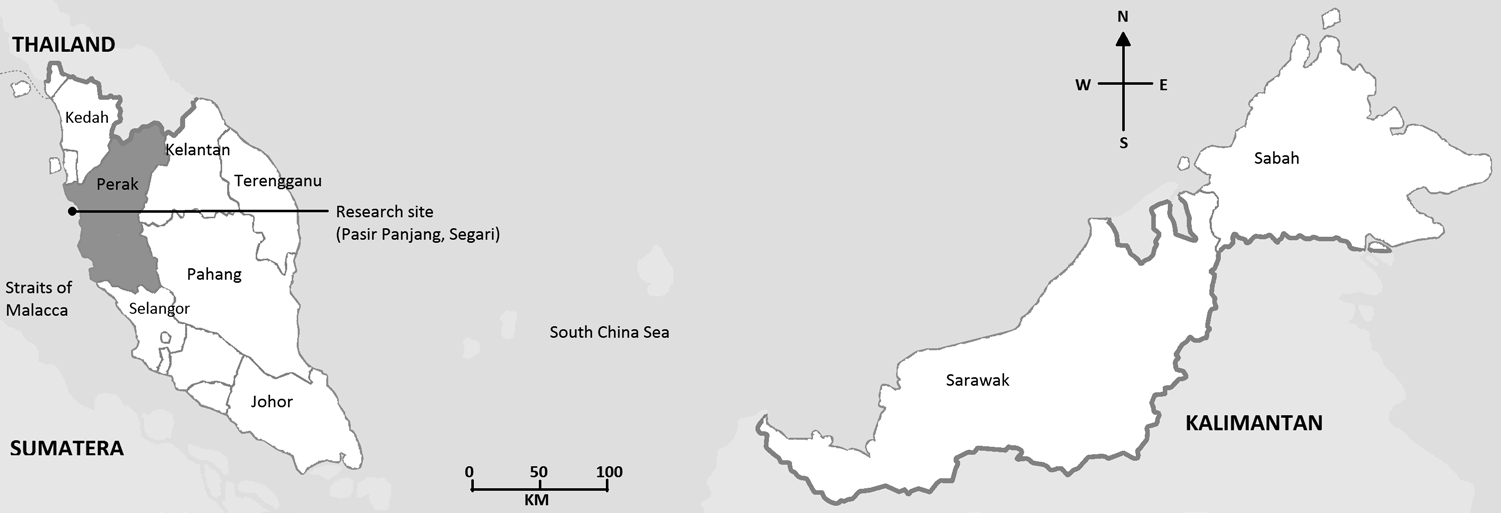
Research site, Pasir Panjang located in Perak, Peninsular Malaysia.

Nesting data from 1998 until 2013 was obtained from the Department of Fisheries with their permission to publish the results. The Department of Fisheries hired local residents and licensed eggs collectors for nocturnal beach monitoring along the beach of Pasir Panjang. The beach patrol was done daily by the local patrollers (1–2 persons), and surveyed along the Pasir Panjang beach (Fig. 1), which is approximately a 5.6 km stretch (Latitude: 4.3465, Longitude: 100.5664) between 21:00 to 06:00 hrs. Nesting activity was observed approximately at 5–10 m distance to avoid distraction to adult turtle during the process. Clutch size was only relocated after the turtle had finished nesting, compacted down the sand using flippers, and returned to the sea. This procedure was conducted to avoid disturbance to the turtle, as the creature is very sensitive to any source of light (i.e. torch light, hand phone light, camera flash) (Chan *et al*. 1999; Wang & Cheng 1999). Artificial lighting (Law *et al*. 2010) and animal disturbance (Maros *et al*. 2003; Donlan *et al*. 2004; Margaret *et al*. 2012; Olgun *et al*. 2016) such as dog, otters, pigs, and foxes also discouraged the nesting of turtles. The standard procedure of beach patrolling in accordance to the Peninsular Malaysian Standard Guidelines of Turtle Management (Sukarno *et al*. 2007), and similar methods can be found in the study of Chen and Cheng (1995); Wang and Cheng (1999); Cheng *et al*. (2009). Identification and tagging method was not implemented for adult green turtle in Perak, similar to marine turtle population in Setiu (Aini Hasanah *et al*. 2014). Adult turtles landed were tagged on both front flippers, and the tagging method is hoped to be included in future as it assists in tracking and identification purpose.

Number of nests and eggs were then counted. Eggs were removed carefully with a minimal orientation from the natural nests to the pile. The pile full with eggs was then transported to the hatchery for immediate incubation. The Department of Fisheries pays RM2 per egg to the licensed eggs collector (Department of Fisheries personal communication). For hatchery monitoring, four staffs (two staffs to work at one time) were hired to look after the eggs incubation inside the hatchery from predator disturbances. The staff also looks after the new emerging hatchlings, and transfers them to a Styrofoam box, to prepare them to be released to the sea between 1–7 days. Survived hatchlings from the hatchery were counted based on the formula: [Total Hatched eggs – Total Dead Hatchlings = Survival hatchlings]. In Perak, morning surveys were performed every day to identify the overlooked or misplaced nest from the previous night. The beach was surveyed from 08:00 to 09:00 hrs. Local residents (i.e. member from Unipro Fishing Club, Segari) occasionally provided information on the nocturnal and daytime nest found. Due to the problems of limited manpower and uncontrolled eggs poaching, all nests found were relocated and *in-situ* method was not performed in Segari.

### Data Analysis (Between 1998 untill 2013)

For the data analysis between 1998 until 2013, record of nesting was unavailable for years 2002–2003, 2004–2005, and 2010–2011 as the data was misplaced and also due to some technical errors (i.e. unreadable data). Due to the above reason, data of nesting, survival hatchlings, and density of visitors for year 2002–2003, 2004–2005, and 2010–2011 were excluded from the analysis. The analysis was only conducted for the survived hatchlings; because the data provided by the Turtle Management Centre was incomplete analyse the hatching success, dead hatchlings, and unhatched eggs.

### Density of Visitors

Density of visitors was obtained from the Turtle Management Centre. Visitors entering the Turtle Management Centre, Segari were required to fill in the record book at the entrance. Total visitors per year were analysed based on the record (from year 1998 until 2013).

### Statistical Analysis

Data was analysed using the SPSS 17.0 version and Microsoft Excel. Spearman’s rank correlation coefficient was used to analyse the correlation between nesting and eggs. Spearman’s Rank coefficient was chosen because the number of nests was small in some years and fluctuated annually. Independent sample *t-*test was performed to test the significant difference between the highest and lowest number of nesting, and eggs per year. One-way ANOVA was used to analyse the uniformity for nesting, eggs, and density of visitors, since the data was not normally distributed, K–S= *p* > 0.05. One sample chi-squared test (i.e., goodness-of-fit test) was used to analyse the uniformity of the survival hatchlings by investigating the number of eggs collected per years.

## RESULTS

### Overall Result of Nesting Distribution

Based on the data obtained from the Department of Fisheries, the number of nesting, eggs, survived hatchlings, and density of visitors in Perak were analysed. According to Table 1, the highest number of nesting with > 300 cumulated nests reportedly occurred in the year of 1998–1999 with 352 nests (mean = 58.67 ± 46.77, median = 48.50), followed by in the year of 2000–2001 with 313 nests (mean = 52.17 ± 33.98, median = 49.00). Meanwhile, year 2008–2009 and 2012–2013 reported to have <200 cumulated nests, with 153 nests (mean = 25.50 ± 13.18, median = 22.50) and 117 nests (mean = 19.50 ± 5.88, median = 19.00). Lastly, year 2006–2007, recorded only 84 cumulated nests, which is the lowest nesting record (mean = 14.00 ± 7.21, median = 16.00). There is no data of nesting for some years due to misplaced original data and unclear data recorded. Due to the incomplete date, accurate nesting result for year 2002–2003, 2004–2005, and 2010–2011 as shown in Table 1 was unable to be added up. The highest record for the sum of nesting per-two months was 135 nesting (occurred in May–June of 1998–1999), followed by 100 nesting (occurred in March–April of 2000–2001), and 92 nesting (occurred in March–April of 1998–1999) (Table 1). The largest sum of eggs collected were 37,177 eggs (in year 1998–1999), followed by 32,231 eggs (in year 2000–2001), 16,033 eggs (in year 2008–2009), 13,794 eggs (in year 2012–2013), and lastly 8,585 (in year 2006–2007). A significant difference between the highest and lowest sum of nesting (1998–1999 and 2006–2007), *t*(5.238) = 2.110, *p* < 0.001, and for sum of eggs (1998–1999 and 2006–2007), *t*(5.335) = 2.122, *p* < 0.001 was identified. Spearman’s correlation was used to test the significant correlation between sum of nesting and sum of eggs per two-years as shown in Table 1. There was a significant correlation between them, Spearman’s rank correlation coefficient (*ρ*) = 0.904, *n* = 30, *p* < 0.001, which proves that there is an increase in the number of eggs by increase the nesting aborted on the beach by turtles.

Meanwhile, the yearly percentage of nesting and eggs is illustrated in Figure 3. The highest nesting was collected in 1999 with 220 nests (21.59%) and 23,622 eggs (21.91%) and the lowest was collected in 2007 with 10 nests (0.98%) and 533 eggs (0.51%). In addition, a significant difference between the highest and lowest number of nesting, *t*(11.125) = 3.245, *p* < 0.001 and eggs, *t*(11.045) = 3.363, *p* < 0.001 as identified. One-way ANOVA test shows that nesting had almost similar distribution across the years [F (9, 6) = 2.342, *p* > 0.05] from 1998 to 2013 (Fig. 3). Similar results were found for eggs distribution per years [F (10, 5) = 1.900, *p* > 0.05].

### Survival Hatchlings

Table 2 illustrates the record of survival hatchlings. The highest survival hatchlings produced was in 2008 with an estimate of 55.03% (or 5,018) from overall 9,119 eggs. In 1999, there was an estimated 51.06% (or 12,061) survival hatchlings produced from 23,622 eggs. Meanwhile in 2013, a total of 14.14% or 2,170 survival hatchlings were produced from 4,916 eggs collected, followed by in 2001, 2000, and 1998 with 41.98% (8,765 survival hatchlings from 20,881 eggs), 41.84% (4,749 survival hatchlings from 11,350 eggs), and 35.57% (4,821 survival hatchlings from 13,555 eggs), respectively. The least percentage was produced in 2007, 2006, and 2012 with 26.20% (145 survival hatchlings from 553 eggs), 25.76 % (2,069 survival hatchlings from 8032 eggs), and 23.33% (2,071 survival hatchlings from 8,878 eggs), respectively. The pattern of survival hatchlings (Table 2) was not uniformly distributed (*χ*^2^ = 160.00, *df* = 100, *p* < 0.001). Overall, 40.98% (44,186) survival hatchlings had been produced from 107,820 eggs collected from 1998–2013.

### Density of Visitors

Fig. 4 shows the density of visitors entering the Turtle Management Centre. Visitors density recorded > 50,000 visits per-two years occurred in 2000–2001 with 55,835 visitors (mean = 9306 ± 1589), followed by 38,941 visitors (mean = 6490 ± 1789), in 2008–2009 with and 30,952 visitors (mean = 5159 ± 1122) in 2012–2013. The least visitor density to the Turtle Management Centre occurred in 2006–2007 and 1998–1999, with <30,000 visitors. Year 2006–2007 recorded 25,616 visitors (mean = 4269 ± 1372) and year 1998–1999 recorded 16,773 visitors (mean = 2796 ± 901). The fluctuating trend of visitors is shown in Fig. 4, which shows ununiformed distribution per-two years, F (7, 40) = 49.152, *p* < 0.001. In the beginning of 1998 to 2001, density of visitors was increasing. The entrance of visitors was stable and fluctuated between 2006 until 2009, before the obvious decline in 2012–2013. Data of visitors were not recorded in year 2002–2003, 2004–2005, and 2010–2011, therefore the graph unable to be plot.

## DISCUSSION

**Figure 2:**
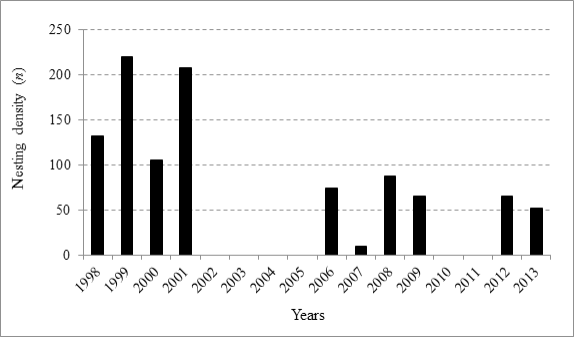
Nesting density per years of *Chelonia mydas* from 1998 to 2013 in Pasir Panjang, Segari, Perak.

**Figure 3:**
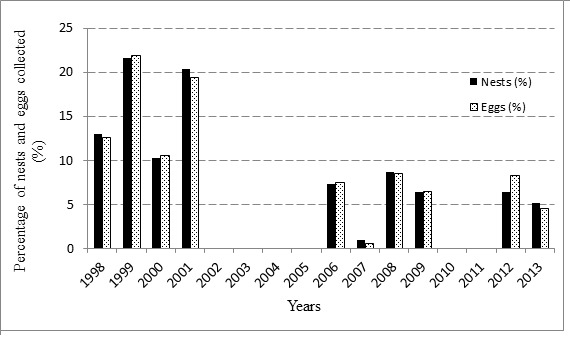
Percentage of nesting and eggs of green turtle (*Chelonia mydas*) from 1998–2013.

The nesting per year varied from 10–220 nesting (Fig. 2). The result is compared with the nesting population in Penang Island [varied from 3–73 nests (Sarahaizad *et al*. 2012a)] and Setiu [28–201 annual nests (Aini Hasanah *et al*. 2014)], and Redang Island [21–687 annual nests (Chan 2010)]. Therefore, it is concluded that green turtle on the west coast of Peninsular Malaysia (Perak and Penang Island) has slightly lesser nesting density than the east coast of Peninsular Malaysia (Setiu and Redang Island, Terengganu). Perak reports an increased yearly nesting than Penang Island; similar average of nesting with Setiu (Terengganu); and, had slightly lower nesting compared to yearly nesting in Redang Island. Beach morphology (Zavaleta-Lizárraga & Morales-Mávil 2013), natural preserved vegetation (Lopez Castro *et al*. 2004; Zare *et al*. 2012; Revuelta *et al*. 2013), type of vegetation (Aini Hasanah *et al*. 2014; Liles *et al.* 2015) and remoteness from human areas (Sarahaizad *et al*. 2012a) are the conditions that attract marine turtles to land. Perak has longer beach (approximately more than 6 km), containing natural and fringed preserved vegetation, remote locations, and exposed to minimal human disturbances, are among the reasons suggested for higher population of green turtle in Perak compared to Penang Island. It is mentioned that olive ridley (*Lepidochelys olivacea*) do nests in Perak (Pusat Pengurusan Penyu, Segari, Perak Darul Ridzuan 2007). Probably due to the missing records, nesting of olive ridley was not found between 1998–2013 in the data provided. Olive ridley might have nested in Pasir Panjang waters, as Penang Island (the adjacent beach) recorded the landing of olive ridley in 2002, 2004, 2005, 2007, 2008, and 2009 (Sarahaizad *et al*. 2012a). Both locations share the same oceans and face the Straits of Malacca. Increasing the manpower and standardising the methods of data recording may solve the problems of overlooked data. In addition, the current situation of poaching rate in Perak is unable to be estimated, as the data was not provided. Therefore, it is hoped that the poaching record could be included in the future.

The pattern of nesting in Perak is estimated to be have a year round nesting (Table 1) similar to the green turtle in Thailand (Yasuda *et al.* 2006). The peak in Perak was estimated between May and June, which is almost similar to graph of peak nesting season as illustrated in Penang Island (Sarahaizad *et al*. 2012a). Since Perak and Penang Island are located facing the Straits of Malacca, the similar peaks nesting might relate to the monsoon in Malaysia. The northeast monsoon in Malaysia occurs end of the year until beginning of the year (November until March) and the winds are stronger than Southwest monsoon that occurs from May until September (MOSTI 2017). Therefore, turtle possibly prefer nesting between May-September, and avoid nesting between November to March during extreme condition of high humidity and low temperature at nesting sites. Previous study proves that humidity at the nesting sites are the critical factors effecting nesting choices (Lopez Castro *et al*. 2004). The similar peak of nesting (May–July) is also reported to occur for hawksbill population in Melaka (Mortimer *et al*. 1993), which is located at the west coast of Peninsular Malaysia. Warmer water temperature during the inter-nesting interval (Hays *et al*. 2002) and temperature of sand (Godley *et al*. 2002) is presumed to promote nesting of marine turtles. In addition, tidal pattern, lunar, and solar are strongly linked and interact with each other as spring tide (highest tide) occurs during the full moon, and neap tide occurs during the during the first and last quarter of the lunar phase (Law *et al.* 2010). Turtle generally nest above the high tidal line (Lopez Castro *et al*. 2004) as emerging at the highest tides will minimise the distance and duration of crawls (Law *et al.* 2010). Even though the preference of nesting related to tidal pattern in Perak was not measured, it is believed that tidal line and lunar factors influence the nesting, because turtle in Penang Island shows a positive sign of emerging during the high tide (Sarahaizad *et al*. 2012b).

The percentage of yearly survival hatchlings fluctuates (23.33% to 55.03%, Table 2). Some years had lower survival hatchlings; due to lack of manpower to control nest from natural predator disturbances that attacks nest inside the hatchery. In Perak, predators that harm lively hatchings are monitor lizard, ghost crab, and red ants. Monitor lizards (*Varanus saluator*) are capable to eat the newly emerged hatchling, attracted from the eggs mucous smells, while groups of red ants bite the lively hatchlings. Ghost crab (*Ocypode* sp*.*) is able to dig a hole, eats the eggs, or drags the lively hatchling into their burrow. Yearly hatchlings survival could be increased with a proper conservation method following the Peninsular Malaysian Standard Guidelines of Turtle Management (Sukarno *et al*. 2007). *In-situ* was not conducted in Perak, therefore relocated or *Ex-situ* nest needs to follow proper guidelines for a maximum hatching success such as incubation at standard nesting depth (suggested to be 65cm depth, Cheng *et al*. 2009). For example, reduce orientation during the eggs handling and incubating relocated eggs immediately (Chan *et al*. 1985; Sukarno *et al*. 2007), avoid sand temperature from exceeding 33°C during incubation as it will decrease hatching success (Matsuzawa *et al*. 2002), and protect nests with plastic mesh to avoid attack from predators (Chan 2010).

In addition, equal hatchling sexes produced from the hatchery’s plot is very important for adult mating process in future when the turtle reaches maturity [approximately 20 years, Davenport (1997)]. Evolutionary theory predicts that male and female offspring should be produced at a 1:1 ratio (Hawkes *et al*. 2013). Higher incubation temperature (> 30°C) tends to produce more females while low incubation temperature (< 28°C) tends to produce more males (Mrosovsky & Yntema 1980). This may be based on a mechanism known as temperature-dependent sex determination, TDS (Hawkes *et al*. 2013) where equal sexes between males and females will be produced. It is suggested the temperature (produced at 50:50 sex ratio) is very close to 29°C (Hays *et al*. 2002). In future, it is hoped that the eggs survivorship results could be recorded separately, between survival hatchling produced from covered hatchery and uncovered hatchery’s plot. Sand temperate monitoring is important for the equal hatchlings sex’s production to ensure sustainability of the species.

Rate of nesting population may show a recovery trend by following proper protection from human hazards such as exploitation of eggs and turtles at nesting site of USA, Japan, Australia, Costa Rica, and Hawaii. (Chaloupka *et al*. 2008) For example, turtle in Hawaii has increased dramatically over 32 years since the protection began in 1978 (Balazs & Chaloupka 2006). Turtle population has not been exposed to human exploitation (turtle harvesting at foraging grounds), harvesting of nesters and eggs, and nesting habitat destruction for a certain time. This resulted in the increase of nesting abundance in Hawaii probably from the increased female nester survival since harvesting of turtles in the foraging grounds was prohibited since the mid-1970s (Balazs & Chaloupka 2006).

**Figure 4:**
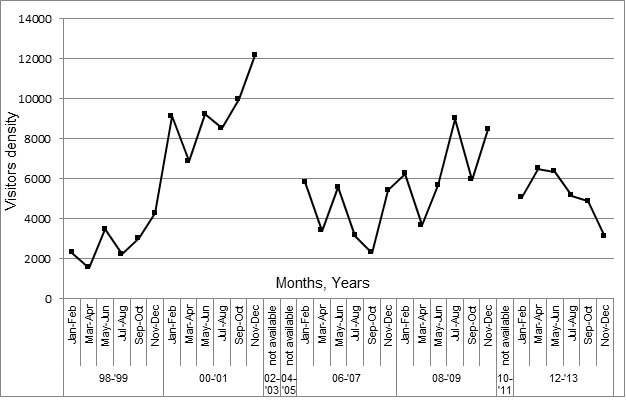
Density of visitors in Pasir Panjang, Segari, Perak.

As illustrated in the graph (Fig. 4), the number of visitors is declining by years. Involvement and support from the locals is important to strengthen the awareness and conservation of marine turtles. In future, an active conservation program by the Department of Fisheries such as conducting hatchling release program, exhibition, and organize international volunteering program involving locals and foreigners such as in Chagar Hutang, Redang Island (Chan 2013) for long term benefits are conducted. From these programs, the society is educated to protect this endangered species. Conservation in Sabah Turtle Island, is one of the successful programs where the nesting sites are fully protected by the Sabah Government. This implementation was due to a recovery trend of marine turtle population in the 1980’s (Chan 2006), resulting from the right conservation methods been forced with the involvement of society in the previous years. Few recommendations are listed below to improvise the management of Turtle Management Centre, Segari, Perak.

1. Current patroller at Pasir Panjang beach (1–2 staffs per night) is not enough to survey the whole stretch of beach as the beach length is approximately >5 km. Therefore, it is recommended to increase the manpower at Pasir Panjang beach for accurate nesting record.
2. Besides Pasir Panjang, nesting occurrence at adjacent beaches such as Sungai Gelam, Tanjung Gemuk, Sungai Puyu, Teluk Cina, Teluk Keling, Tanah Runtuh, and Tanjung Hantu were not properly recorded. Lack of staff resulted in overlooked nesting as not all the beaches were surveyed every night. These beaches were occasionally surveyed (once in a month) based on local residents’ report. It is recommnded for sufficient patrollers to be placed per beach as it is currently done in many turtle conservation centres in Malaysia [i.e., in Melaka (Mortimer *et al*. 1993); Penang Island (Sarahaizad & Shahrul Anuar 2015); Terengganu (Aini Hasanah *et al*. 2014)].
3. In future, it is suggested to include breeding biology data such as digging attempts, false crawls, nest placement, emergence hour, and morphological characteristics. This data records may be referred to Kerachut Turtle Conservation Centre in Penang Island (Sarahaizad *et al*. 2012b; Sarahaizad & Shahrul Anuar 2015).
4. As eggs were relocated and incubated in hatchery, the division of eggs survivorship is important to know the eggs incubation status. The eggs survivorship can be divided into; survival hatchlings, hatched eggs, unhatched eggs, dead hatchlings, embryonic eggs, and predated eggs. These formulas of eggs survivorship is referred to Chen and Cheng (1995), and Olgun *et al*. (2016).
5. Garbage (i.e. plastic, styrofoam, plastic container, boxes, papers, glasses) that is obviously seen along the Pasir Panjang beach might affect the nesting due to pollution at nesting ground and feeding oceans. Regular beach cleanliness activity is able to reduce pollution at nesting ground.
6. It is hoped that adult landing could be tagged in future, so the landed adults are estimated. In addition, the migration of turtles may be detected, whether the green turtle in Perak have migrated to Penang Island, or vice versa. This co-operation can be made, as both nesting locations are located at the northern region of Peninsular Malaysia.
7. Juvenile turtles (approximately more than 7 years age) were found to be kept inside the pool with no artificial nesting ground built for them to land and perform nesting. These turtles may face difficulty to survive if released to the sea. The natural navigation may be interrupted since they were kept in the pool as soon as they were hatched. Marine turtles will move to waters in closer vicinity to nesting beaches (Bowen *et al*. 2005) and ready to lay eggs as they approach maturity. Therefore, it is suggested for the artificial nesting ground to be included in the pool for them to lay eggs. This may be referred to the example such as in Port of Nagoya Public Aquarium, where both males and females will mate inside the aquarium, and lay eggs at the artificial nesting ground (Sarahaizad personal observation). This project was one of the successful project in Japan.
8. Lastly, it is suggested to avoid splitting the eggs into smaller sizes for eggs incubation, as it reduces the hatchling energy reserved when they enter the sea (Rusli *et al*. 2016). This may have an implication to the hatchling’s survival. Whole eggs incubation with a proper nesting without splitting the eggs is recommended, as it is also able to produce high rates of hatching success (Sarahaizad & Shahrul Anuar 2014).

## CONCLUSION

In this paper, the nesting density, eggs, survival hatchlings, and density of visitors in Segari Perak between 1998 until 2013 was estimated. This publication provides a basic knowledge of nesting population in Perak, and can be used as a reference for upcoming studies. By updating the current nesting status, the results might be helpful for purpose of conservation. The ideas given in this paper may provide benefit for the reason to upgrade the management of Turtle Management Centre and Department of Fisheries in future. A serious conservation in Perak is needed to prevent the species from declining by involving local and international participants.

## Figures and Tables

**Table 1:** Sum of nesting and eggs of green turtle, *Chelonia mydas* in Perak.

Years	Months	Sum of nesting	Mean ± SD (*n* = 6*)*	Overall sum of nesting	Median (*n* = 6)	Sum of eggs	Overall sum of eggs
1998–1999	Jan–Feb	17	58.67 ± 46.77	352	48.50	1635	37177
	Mar–Apr	92				10135	
	May–Jun	135				14131	
	Jul–Aug	80				8181	
	Sep–Oct	16				1607	
	Nov–Dec	12				1488	
2000–2001	Jan–Feb	42	52.17 ± 33.98	313	49.00	4706	32231
	Mar–Apr	100				10317	
	May–Jun	89				8862	
	Jul–Aug	56				5507	
	Sep–Oct	20				2010	
	Nov–Dec	6				829	
2002–2003	Not available						
2004–2005	Not available						
2006–2007	Jan–Feb	17	14.00 ± 7.21	84	16.00	1911	8585
	Mar–Apr	20				2255	
	May–Jun	23				2536	
	Jul–Aug	15				1374	
	Sep–Oct	3				301	
	Nov–Dec	6				208	
2008–2009	Jan–Feb	19	25.50 ± 13.18	153	22.50	1698	16033
	Mar–Apr	26				2610	
	May–Jun	51				5583	
	Jul–Aug	30				3174	
	Sep–Oct	9				938	
	Nov–Dec	18				2030	
2010–2011	Not available						
2012–2013	Jan–Feb	13	19.50 ± 5.88	117	19.00	3203	13794
	Mar–Apr	26				3222	
	May–Jun	26				3204	
	Jul–Aug	24				1568	
	Sep–Oct	14				2047	
	Nov–Dec	14				550	

**Table 2:** Number and percentage of survival hatchlings (1998–2013) from Turtle Management Centre, Perak.

Years	Total eggs	Survival hatchlings	%
1998	13,555	4,821	35.57
1999	23,622	12,061	51.06
2000	11,350	4,749	41.84
2001	20,881	8,765	41.98
2002	0	0	0.00
2003	0	0	0.00
2004	0	0	0.00
2005	0	0	0.00
2006	8,032	2,069	25.76
2007	553	145	26.20
2008	9,119	5,018	55.03
2009	6,914	2,317	33.51
2010	0	0	0.00
2011	0	0	0.00
2012	8,878	2,071	23.33
2013	4,916	2,170	44.14
Total	107,820	44,186	40.98
